# Trauma-Informed Care in an 8-Year-Old With Neurodivergence and Childhood Adversity: Clinical Reflections and the Impact of Secondary Trauma in CAMHS

**DOI:** 10.1192/bjo.2025.10747

**Published:** 2025-06-20

**Authors:** Yasir Mateen, Mariam Khurshid

**Affiliations:** 1 South West Yorkshire Partnership NHS Foundation Trust, Huddersfield, United Kingdom; 2 South West Yorkshire Partnership NHS Foundation Trust, Halifax, United Kingdom

## Abstract

**Aims:** Children with neurodevelopmental conditions, particularly ADHD, often face heightened vulnerability to adverse childhood experiences (ACEs), shaping their mental health trajectories. Trauma-informed care (TIC) provides a structured framework for understanding and addressing these vulnerabilities within clinical practice. Additionally, working with trauma-exposed children places significant psychological demands on mental health professionals, contributing to secondary traumatic stress (STS) and burnout. This study presents a complex case from CAMHS, illustrating the intersection of ADHD, trauma, and systemic care challenges, alongside an exploration of clinician well-being within trauma-intensive settings.

**Methods:** A single case study approach was employed, utilizing Bronfenbrenner’s Ecological Systems Theory to analyse the interplay between individual, familial, and systemic factors affecting an 8-year-old child’s mental health. A review of trauma-related clinician distress was conducted, drawing from literature on secondary trauma and workforce resilience within CAMHS services.

**Results:** The case study underscores the bidirectional relationship between trauma and ADHD. Despite medical and educational interventions, ongoing adversity exacerbated symptom severity, necessitating a trauma-informed, whole-system approach. Additionally, clinicians working within trauma-intensive environments demonstrate significant risks for STS and burnout, emphasizing the need for structured trauma stewardship interventions.

A dual-focused approach in CAMHS – addressing both patient needs and clinician well-being – is critical for sustaining high-quality care. Conceptual frameworks such as the Biopsychosocial Model and the Boundary Seesaw Model provide strategies to navigate the competing demands of care provision and workforce resilience. Reflective practice, clinical supervision, and organizational support emerged as critical factors in maintaining a sustainable mental health workforce.

**Conclusion:** Trauma-informed care is essential for managing neurodevelopmental conditions within adversity-laden contexts. Optimizing CAMHS services requires integrating trauma-responsive strategies at both patient care and workforce levels to ensure sustainable, high-quality mental health support. This study underscores the urgency of workforce resilience policies within trauma-informed mental health systems.

